# Rapid Antibody Selection Using Surface Plasmon Resonance for High-Speed and Sensitive Hazelnut Lateral Flow Prototypes

**DOI:** 10.3390/bios8040130

**Published:** 2018-12-14

**Authors:** Georgina M.S. Ross, Maria G.E.G. Bremer, Jan H. Wichers, Aart van Amerongen, Michel W.F. Nielen

**Affiliations:** 1 RIKILT, Wageningen University & Research. P.O Box 230, 6700 AE Wageningen, The Netherlands; monique.bremer@wur.nl (M.G.E.G.B.); michel.nielen@wur.nl (M.W.F.N.); 2 Wageningen Food & Biobased Research, BioSensing & Diagnostics, Wageningen University & Research, P.O Box 17, 6700 AA Wageningen, The Netherlands; jan.wichers@wur.nl (J.H.W.); aart.vanamerongen@wur.nl (A.v.A.); 3 Wageningen University, Laboratory of Organic Chemistry, Helix Building 124, Stippeneng 4, 6708 WE Wageningen, The Netherlands

**Keywords:** surface plasmon resonance, high-speed lateral flow immunoassay, food allergen, carbon nanoparticles, antibody selection, smartphone detection

## Abstract

Lateral Flow Immunoassays (LFIAs) allow for rapid, low-cost, screening of many biomolecules such as food allergens. Despite being classified as rapid tests, many LFIAs take 10–20 min to complete. For a really high-speed LFIA, it is necessary to assess antibody association kinetics. By using a label-free optical technique such as Surface Plasmon Resonance (SPR), it is possible to screen crude monoclonal antibody (mAb) preparations for their association rates against a target. Herein, we describe an SPR-based method for screening and selecting crude anti-hazelnut antibodies based on their relative association rates, cross reactivity and sandwich pairing capabilities, for subsequent application in a rapid ligand binding assay. Thanks to the SPR selection process, only the fast mAb (F-50-6B12) and the slow (S-50-5H9) mAb needed purification for labelling with carbon nanoparticles to exploit high-speed LFIA prototypes. The kinetics observed in SPR were reflected in LFIA, with the test line appearing within 30 s, almost two times faster when F-50-6B12 was used, compared with S-50-5H9. Additionally, the LFIAs have demonstrated their future applicability to real life samples by detecting hazelnut in the sub-ppm range in a cookie matrix. Finally, these LFIAs not only provide a qualitative result when read visually, but also generate semi-quantitative data when exploiting freely downloadable smartphone apps.

## 1. Introduction

Lateral flow immunoassay (LFIA) is a rapid technique which relies on the fast interaction between an antibody and a target antigen [1]. These devices have experienced a surge in popularity in the medical and food safety fields, since their birth as home-pregnancy tests [2]. It is preferred for LFIAs to use purified, fast, specific and properly characterized antibodies [3]. Although LFIAs are classified as a rapid method, they still require 10–20 min to complete [4]. In order to create high-speed LFIAs, it is necessary to test the antibody rate of association towards the target analyte, as well as use a nitrocellulose membrane with a high flow rate. Traditional antibody selection techniques, such as enzyme linked immunosorbent assay (ELISA) and western blot, do not necessarily convert well into LFIAs due to the much faster rate of kinetics in LFIA [5]. As trends move toward rapid on-site testing, with consumer-friendly tests such as LFIA and smartphone-based readout systems, the need for antibodies with rapid association towards their target becomes more apparent [4]. In addition to requiring fast antibodies, it is necessary to have a rational way of quickly comparing and selecting such antibodies. One way of speeding up the antibody screening and LFIA prototyping process is to use a label-free biosensor to compare relative antibody-antigen association binding speeds to facilitate the selection process [6,7,8]. 

Surface plasmon resonance (SPR) is one such technique. SPR allows label-free, optical monitoring of important kinetic information, such as the association and dissociation rates of antibodies, in real time [9]. Using SPR it is possible to screen crude antibodies. Herein, the term crude refers to: a mixture of un-purified, cell culture media with variable specific antibody concentrations. Screening crude monoclonal antibodies (mAbs) saves time and money in comparison with first purifying a panel of mAbs and then testing them all for application in LFIA [10]. Previously, true kinetic studies have been carried out to select antibodies based on their affinities, association and dissociation rates, for application in a direct SPR biosensor [11]. In the medical sector there is interest in screening and ranking hybridomas, hybrid cells formed from the antibody producing spleen cell of an immunized animal fused with a myeloma cell, for their affinities. However, studies in the literature have not yet focused on the ranking and selection of antibodies based on their association rates towards food allergens, for application in rapid ligand-binding assays such as LFIA [6,12,13]. Current antibody selection processes using SPR are affinity based and the antibodies are screened against purified analytes. By contrast, in this study, an unpurified hazelnut extract, which is a complex mixture of heterogeneous proteins of various molecular weights, is the target analyte [14]. 

When developing sandwich format assays for large molecular weight proteins (e.g., food allergens) it is essential to select appropriate antibody pairs for the capture and detection of the target analyte [15]. Hazelnut has been selected as the target for this study, as hazelnut is considered the most prevalent tree nut allergy in Europe [16]. Sandwich pairs are antibodies that are capable of simultaneously binding an antigen. Pre-matched antibody pairs can be purchased from commercial vendors which can save time and resources, or they can be selected through sandwich pairing experiments [17]. These pairs are often found using ELISA. However, the results obtained in ELISA do not always predict how the antibodies will perform in LFIA [3]. Alternatively, antibody pairs can be determined by using a half-stick format LFIA [18]. Pairwise selection can also be achieved using biosensors by epitope binning [19]. This process assesses whether antibodies bind to overlapping epitopes on the target antigen, or whether they are capable of binding to different epitopes [20]. Using SPR to select antibody pairs for use in LFIA saves time and can be largely automated for screening large antibody panels for their pairs [20]. 

To the best of the authors’ knowledge, this is the first example of using SPR as a screening method for selection of high-quality antibodies from crude samples for application in LFIA. SPR has been utilized for selection of purified mAbs for these characteristics, illuminating its importance as a selection tool in this sector [18]. A batch screening method was designed using an FC-specific anti-mouse IgG (FC-IgG) immobilised onto an SPR chip. The FC-IgG captures the anti-hazelnut antibodies of interest on the surface. Subsequently, hazelnut protein extract is injected and the binding between the antibody and hazelnut is monitored. Using an FC-IgG surface offers on chip affinity purification of the crude sample, as it captures the crude anti-hazelnut antibodies in their FC region. This allows the captured crude antibodies to be uniformly distributed, in the assumed correct orientation, without any compromise of their biological activity [21]. Furthermore, using an FC-IgG expedites the regeneration of the chip surface between each cycle for screening subsequent antibodies. Following normalization, to compensate for differences in specific antibody concentrations in crude samples, a visual assessment can be made to compare the relative association rates of each mAb towards hazelnut. The un-purified antibodies can then be ranked based on their fast association, sensitivity, specificity and sandwich pairing. As a result, fast (F) and slow (S) antibody pairs selected by SPR were used to develop a carbon nanoparticle-based LFIA system, to identify whether similar kinetics could be observed in LFIA as was seen in SPR. Amorphous carbon nanoparticles are excellent labels in LFIA as they are easy and low-cost to prepare; have high signal to background contrast, making them easier to read with the naked eye; and can allow for increased sensitivity compared with other labels [22]. Even lower LOD’s might be achieved for carbon nanoparticle-labelled LFIAs by using a flatbed scanner to determine grey pixel values. An alternative, more consumer-orientated method is to use smartphone apps to determine RGB/CMYK values of the test line region of the LFIAs and to convert these to LAB (where L is Luminance and A and B are color channels) values. Whilst RGB (red, green, blue) and CMYK (cyan, magenta, yellow, key) values are device dependent, LAB values provide device independent information about the darkness/lightness of a selected region of an image [23]. In this way a calibration curve of LAB color values against allergen concentration (ppm) can be plotted for semi-quantification of LFIA results. Furthermore, there are currently no food allergen LFIAs that apply carbon nanoparticles, exemplifying the label novelty in this field [24]. The LFIA prototypes developed were compared based on their speed and sensitivity and applied to a real food matrix of cookies as a proof-of-concept. Cookies have been selected for a matrix as a 2018 report determined that products such as cookies, chocolate and bread are responsible for the majority of accidental allergic reactions [25]. Finally, the LFIAs were semi-quantified by a smartphone using freely downloadable color analysis apps. 

## 2. Materials and Methods

### 2.1. Equipment

All SPR experiments were carried out using a BIACORE 3000 (GE Healthcare, Uppsala, Sweden). An EL x 808 BioSPX Microplate Reader was used for the determination of the Bicinchoninic acid (BCA) results (Beun De Ronde, Abcoude, The Netherlands). A NanoDrop ND-3300 (Isogen Life Sciences, De Meern, The Netherlands) or the DeNovix DS-11 spectrophotometer (DeNovix, Wilmington, DE, USA) was used for all other protein quantifications. A Braun Turbo 600 W Food Processor (Kronberg im Taunus, Germany) was used for homogenizing the food samples. All food extracts were filtered through low-binding syringe filters (5 to 0.45 µm; Pall Life Sciences, Portsmouth, UK). The LFIA strips were sprayed using a Linomat IV TLC-spotter (CAMAG, Berlin, Germany). The CM4000 BioDot Guillotine (Biodot Inc., Irvine, CA, USA) was used to cut the strips. A Bioruptor Plus Diagenode (Diagenode SA, Seraing, Belgium) was used to sonicate the carbon nanoparticle suspensions. All smartphone video recordings and photos were taken using a Google Pixel 2 XL (Google, Mountain View, CA, USA). All smartphone-based color detection was accomplished using ‘RGB Color Detector’ (version 1.0.35, The Programmer; Google Play Store) and color conversions using ‘Nix Pro Color Sensor’ (version 1.28; Nix Sensor Ltd., Hamilton, ON, Canada; Google Play Store). 

### 2.2. Chemicals & Reagents 

The SPR experiments were carried out using carboxymethylated dextran sensor chips (CM5), HBS-EP buffer (pH 7.4, consisting of 10 mM 4-(2-hydroxyethyl)piperazine-1-ethanesulfonic acid, 150 mM sodium chloride, 3 mM ethyldiaminetetraacetic acid, 0.005% *v*/*v* surfactant polysorbate 20), an amine coupling kit (containing: 0.1 M *N*-hydroxysuccinimide (NHS), 0.4 M 1-ethyl-3-(3-dimethylaminopropyl)carbodiimide hydrochloride (EDC) and 1 M ethanolamine hydrochloride (pH 8.4)), all purchased from GE Healthcare (Uppsala, Sweden). Bovine serum albumin (BSA) was purchased from Sigma-Aldrich (Zwijndrecht, The Netherlands). Analysis of all SPR results was performed using the BiaEvaluation software (Biacore, Uppsala, Sweden). 

The washing buffer (WB) was composed of 5 mM borate buffer (BB) (pH 8.8) diluted from a mixture of 100 mM sodium tetraborate (VWR, Leuven, Belgium) and 100 mM boric acid (Merck, Darmstadt, Germany), and bovine serum albumin (BSA) was added to a final concentration of 1% (*w*/*v*). The storage buffer (SB) consisted of 100 mM BB containing BSA to a final concentration of 1% (*w*/*v*). The running buffer (RB) was prepared by adding 1% BSA (*w*/*v*) and 0.05% Tween-20 (*v*/*v*) (Merck, Darmstadt, Germany) to 100 mM BB. TRIS-buffered saline (TBS; pH 8.2) was prepared from 20 mM TRIS (Duchefa Biochemie, Haarlem, The Netherlands) and 300 mM NaCl (Merck, Darmstadt, Germany). Phosphate-buffered saline (PBS) pH 7.4 was purchased from Sigma-Aldrich (Sigma-Aldrich, St Louis, MO, USA). The BCA reagents were purchased from Pierce (Rockford, IL, USA). All solutions were prepared with MQ water from a MilliQ-system (>18.2 MΩ/cm) purchased from Millipore (Burlington, MA, USA). ‘Spezial Schwartz 4’ carbon nanoparticles were purchased from Degussa AG (Frankfurt, Germany). Goat anti-mouse IgG Fc specific antibody in PBS (2.4 mg/mL) used in the SPR study was purchased from ThermoFisher Scientific (Landsmeer, The Netherlands). Goat anti-mouse IgG in PBS (pH 7.6) (1.2 mg/mL; AffiniPure F(ab’)_2_ Fragment GAM IgG Fcγ) used for spraying LFIA control lines was purchased from Jackson Immunoresearch Laboratories Inc (Sanbio, Uden, The Netherlands). All other antibodies were developed by RIKILT, Wageningen University & Research (Wageningen, The Netherlands), according to the procedure described in [26,27]. In short, the antibody panel listed in Table 1 was produced by immunizing mice with 50 µg extracted hazelnut (mixed) protein, with booster immunizations containing 25 µg extracted hazelnut protein. Antibodies selected for LFIA were purified using a HiTrap Protein G column (GE Healthcare, Uppsala, Sweden). Briefly, antibodies were collected from 1 L of raw cell culture media by ammonium sulphate precipitation and subsequent affinity chromatography purification. Following this method, around 15–20 mg of purified antibodies was obtained from 1 L of raw cell culture medium. 

### 2.3. Allergen Extractions

Certified standardized reference materials for food allergens are not commercially available and so antigen standards require in-house preparation. Allergen extracts were made from a ‘blank’ matrix of organic whole meal digestive biscuits (containing: flour, palm oil, sugar, barley malt extract, sodium bicarbonate, ammonium bicarbonate, salt; Dove’s Farm Organic Whole meal Digestive Biscuits; Dove’s Farm, Berkshire, UK), from hazelnut cookies (TimeOut Hazelnoot Granenbiscuits containing: 10% hazelnut, egg, milk & sesame; Albert Heijn, The Netherlands) and from hazelnuts, pecan nuts, pistachio nuts, brazil nuts, peanuts, cashew nuts, almonds, walnuts and macadamia nuts, which were all purchased from a local supermarket. All extracts were filtered through a series (5 µm, 1.2 µm, 0.45 µm) of low protein-binding syringe filters. For the SPR study, whole raw hazelnuts were frozen at −80 ^°^C for 4 h. The frozen hazelnuts were homogenized to a fine powder using a commercial hand blender. The protein was extracted by adding 10 mL of heated TBS buffer per gram of ground hazelnut. The mixture was vortexed for 30 s before rotating end-over-end for 30 min at 37 ^°^C. The solution was centrifuged at room temperature for 15 min at 4000× *g*. The resulting liquid phase was filtered through a series of low protein-binding syringe filters. Total protein concentrations were determined according to the BCA protein assay using BSA as the standard. All hazelnut protein extracts were aliquoted and stored at −20 ^°^C until use. For the cross-reactivity study, a universal allergen extraction procedure was applied that can be used to simultaneously extract multiple different food allergens. Extracts were made from hazelnut, peanut, pecan, pistachio, walnut, brazil nut, macadamia nut, almond and cashew following the method described by Raz [28]. Briefly, nuts were homogenized using a Braun Turbo 600 W Food Processor, and 0.25 g sample portions were weighed out. Twenty-five millilitres of PBS (pH 7.4) was added to the ground samples and incubated at room temperature for 1 h. Following incubation, extracts were centrifuged at 3220× *g* for 20 min. The extracts were then filtered through a series of low protein-binding syringe filters, aliquoted and stored at −20 ^°^C until use. The same procedure was applied for the matrix extraction of the ‘Blank’ matrix and hazelnut cookies but using a 2.5 g ground food in 25 mL PBS. Total protein contents of all allergen/matrix extracts were determined using the NanoDrop. 

### 2.4. Biosensor Chip Preparation

A standard amine coupling procedure was applied at 25 ^°^C to immobilize the Fc-Specific IgG (FC-IgG) onto the CM5 surface. Immobilization pH scouting for coupling of FC-IgG to CM5 chip was performed. The FC-IgG was diluted to 20 µg/mL in 10 mM sodium acetate of varying pH’s and tested using the pH scouting wizard in the Biacore 3000 control software (Uppsala, Sweden). A high immobilization level was reached at pH 5.5, so sodium acetate pH 5.5 was selected as the immobilization buffer in the following procedure. 

The four flow channels, clamped against the carboxylmethylated (CM) dextran chip surface, were simultaneously activated by injecting 35 µL of a mixture of EDC and NHS (1:1 *v*/*v*) at a flow rate of 5 µL/min. Then, FC-IgG diluted (20 µg/mL) in coupling buffer (10 mM sodium acetate, pH 5.5) was injected in flow cells 2–4, and FC-IgG was attached to the activated CM-dextran surface via its exposed primary amine groups. Flow cell 1 was used as a reference channel and was left blank and was only activated by EDC/NHS. The coupling was followed by blocking the remaining active ionic groups in all flow cells with ethanolamine (1 M) preventing electrostatic interactions with the CM-dextran surface. Around 10,000 RU of FC-IgG was immobilized in each channel (2–4) using this method; this high level was aimed for in order to properly cover the chip surface with FC-IgG for the subsequent capture of the specific anti-hazelnut mAbs of interest.

### 2.5. Crude Antibody Screening Assay

The screening analysis was performed at 25 ^°^C using HBS-EP (pH 7.4) as the screening buffer. The crude antibodies were diluted 1/20 in the screening buffer. The hazelnut protein extract was diluted to 20 ppm in the screening buffer. Twenty microliters of each crude antibody dilution was injected at a flow rate of 20 µL per minute for capture. These flow conditions were selected to more accurately reflect the fast flow kinetics observed in LFIAs. Subsequently, 20 µL of 20 ppm hazelnut extract was injected at a flow rate of 20 µL per minute. The surfaces were immediately regenerated with 2 pulses of 5 µL, 5 mM NaOH to return the biosensor signal to baseline [29]. A range of different regeneration conditions were tested, including glycine, HCl and different strengths, volumes and flow rates of NaOH. Of all the tested regeneration conditions, 2 short NaOH pulses were found to be the most appropriate for removing both strong and weak binders whilst minimising FC-IgG surface deterioration, and these were applied as the standard regeneration conditions. 

Using the Biaevaluation software (Biacore, Uppsala, Sweden), the whole sensorgrams for each crude antibody capture and antigen binding cycle were superimposed. As the antigen in this study is comprised of heterogeneous proteins, the curves do not conform to Langmuir binding models. Therefore, as this study is focused on a rapid screening process, a full kinetic curve fitting was not performed. The sensorgrams were aligned on the x-axis at the hazelnut antigen injection point. A snapshot of the relevant part of the sensorgram, containing the hazelnut association and dissociation data, was made in the software. The sensorgrams were double referenced, first by using flow cell 1 as a blank reference channel for buffer signal subtraction and subsequently by normalizing the hazelnut response by dividing the antigen response by the corresponding crude antibody capture level, as described in [11]. All sensorgrams were y-axis zeroed to baseline. After data processing and removal of the FC-IgG capture curve and the regeneration peaks, a visual assessment of the association rates of each antibody towards hazelnut could be achieved. The visual assessment of the steepness of the association curves for the crude antibodies toward hazelnut was confirmed using the slope analysis function in Microsoft Excel. 

### 2.6. Cross-Reactivity Testing 

Total protein extracts from tree nuts and peanut (in PBS) were protein content determined using the NanoDrop and then were diluted to 100 ppm in HBS-EP. Three different antibodies were captured by the FC-IgG surface, in individual flow cells, at a flow rate of 20 µL per minute. During the first cycle, 20 ppm hazelnut extract was injected as a control to monitor the binding response of these crude antibodies towards hazelnut. Following this, the surface was regenerated with the standard regeneration conditions. Subsequently, the same antibodies were re-captured and 20 µL of one of the other tree nut/peanut protein extracts was injected over the antibodies using the same flow conditions. Following this, the surface was regenerated using 1 or 2 pulses of 5 mM NaOH, depending on the extent of tree nut/peanut binding. The procedure was repeated for all of the tree nut/peanut extracts. 

### 2.7. Sandwich Pairing Assay

Twenty microliters of each of three antibodies was captured in individual flow cells at a flow rate of 20 µL per minute. Next, 20 µL of 20 ppm hazelnut extract was injected over all flow cells simultaneously at flow of 20 µL per minute. Subsequently, 20 µL of one crude antibody was injected over all three flow cells, generating data for one antibody against itself and against two other antibodies. Following this, the surface was regenerated with standard conditions to return the signal to baseline. 

### 2.8. Labelling with Carbon Black Nanoparticles 

A 1% suspension of carbon nanoparticles was prepared by adding 1 mL of MilliQ Water (MQ) to 10 mg carbon and sonicating for 10 min. The resulting 1% carbon suspension was diluted five times in 5 mM BB (pH 8.8) to obtain a 0.2% suspension, which was then sonicated for a further 5 min. Next, 350 µg of purified anti-hazelnut antibody was added per 1 mL of 0.2% carbon suspension and stirred overnight at 4 ^°^C. The suspension was divided into two aliquots and 500 µL of WB was added to each and centrifuged for 15 min at 13,636× *g* at 4 ^°^C. Following this, the supernatants were removed and the pellets re-suspended in WB, this process was repeated 3 times. After the final wash, the supernatants were discarded, and the pellets were pooled together with 1 mL storage buffer and stored at 4 ^°^C until use. Scanning electron microscopy (SEM) images of F-50-6B12-carbon nanoparticle suspension can be seen in the Supplementary Material (Figure S1). 

### 2.9. Lateral Flow Immunoassay 

#### 2.9.1. Preparation of Lateral Flow Immunoassay Prototype 

Lateral flow strips were manufactured using nitrocellulose (NC) membranes (HiFlow Plus HF13502; Millipore, Carrigtwohill, Co. Cork, Ireland) cut to approximately 2.5 cm in length; an SEM image of the NC membrane can be seen in the Supplementary Material (Figure S2). The NC membrane was secured on a plastic backing (G & L, San Jose, CA, USA), with 4.5 cm of absorbent pad (Schleicher & Schuell, Dassel, Germany) overlapping one end of the NC. Four LFIAs were prepared for each antibody, with different antibody concentrations dispensed onto the test line to determine the optimum conditions. A TLC spotter was used to dispense the test line (the anti-hazelnut antibody at 0.2 mg/mL, 0.15 mg/mL, 0.1 mg/mL or 0.05 mg/mL) at 1.2 cm and the control line (Goat anti-mouse Fab Fragment at 0.1 mg/mL) at 1.5 cm from the sample application end of LFIA. The TLC spotter used 1 µL of antibody per 5 mm wide strip, at a speed of 15 µL per second. The membranes were allowed to dry at room temperature for 30 min. Finally, 5-mm-wide strips were cut using the BioDot Guillotine CM4000 (Biodot Inc., Irvine, CA, USA) and were packaged in aluminium pouches with silica desiccation packs, heat-sealed and stored at room temperature until future use. 

#### 2.9.2. Lateral Flow Immunoassay: Limit of Detection 

First, the visual limit of detection (LOD) of the strip tests was determined using a decreasing concentration of hazelnut protein extract diluted in PBS. Herein, the visual LOD is defined as the lowest concentration of total hazelnut protein capable of resulting in the appearance of a test line. Both strip batches had the same amount of purified anti-hazelnut antibody immobilized on the test line, and both sets of carbon nanoparticle labelled mAbs had 350 µg of antibody immobilized per mL of carbon so that a fair comparison could be made between the two sets of antibodies. For dipstick analysis, a strip test was placed in a well of low binding microtiter plate containing 100 µL running buffer (RB), 1 µL carbon-antibody conjugate and 1 µL hazelnut extract (dilution range: 100, 50, 25, 10, 5, 2.5, 1, 0.5, 0.25, 0.1, 0 ppm) and was allowed to run for 5 min. Subsequently, the visual LOD of the dipsticks in a spiked commodity was determined. To test for matrix LOD’s, total hazelnut protein extract was spiked into a blank cookie extract in the range of 100 ppm to 0.5 ppm (100 ppm, 50 ppm, 25 ppm, 10 ppm, 5ppm, 2.5 ppm, 1 ppm, 0.5 ppm, 0 ppm). The testing procedure was the same as that described above. Additional matrix LOD determinations were made using 50 µL RB, 50 µL spiked commodity and 1 µL carbon conjugated-mAb in order to reduce further dilution that was caused by adding 100 µL of running buffer to 1 µL of sample. In order to establish the real life applicability of the optimal LFIA, the real life matrix of a hazelnut cookie extract was also tested (1 µL sample in 100 µL RB) spiked into a decreasing dilution in a blank cookie extract in the range of 1:1 to 1:1,000,000. 

#### 2.9.3. Lateral Flow Immunoassay: Test Line Kinetics 

To compare the antibody to hazelnut association rates in LFIA, it is necessary to time the appearance of the test line. The strips were tested by inserting a test strip into a microwell containing 100 µL RB, 1 µL carbon-mAb and 1 µL of 50 ppm hazelnut protein extract (in PBS). A higher concentration of hazelnut extract was used for the kinetic study, as a higher analyte level results in the appearance of a darker line with a high contrast, making it easy to visualize the line as soon as it forms. Instead of allowing the strips to run for 5 min, as soon as a test line appeared on the strip, this time was recorded. The kinetic experiments were repeated multiple times (*n* = 8) and were assessed visually and by smartphone video recording for the test line formations. 

#### 2.9.4. Semi-quantitative Smartphone Lateral Flow Readout 

To obtain RGB/CMYK color values, each LFIA in a calibration range (100 ppm, 50 ppm, 25 ppm, 10 ppm, 5 ppm, 2.5 ppm, 1 ppm, 0.5 ppm, 0 ppm) was analysed, with ‘RGB Color Detector’ (version 1.035), by selecting a region of interest in the LFIA test line area using the crosshair function. To obtain fair color values, values were averaged from three distinct points on the test line of the strips (*n* = 3). Color values were also taken from the background (below the test line) to normalize the results. The ‘Nix Pro Color’ (version 1.28) sensor allows conversion between multiple different color spaces. Therefore, when plugging the RGB or CMYK values obtained in ‘RGB Color Detector’ into the ‘Nix Pro Color’ sensor, it is possible to select a conversion to LAB (or cieLAB) color space. Using the obtained LAB values, a calibration curve was plotted for LAB values vs hazelnut extract spiked into blank cookie extract using an ordinary spreadsheet program. 

## 3. Results

### 3.1. SPR Crude Antibody Screening Assay 

**Figure 1:**
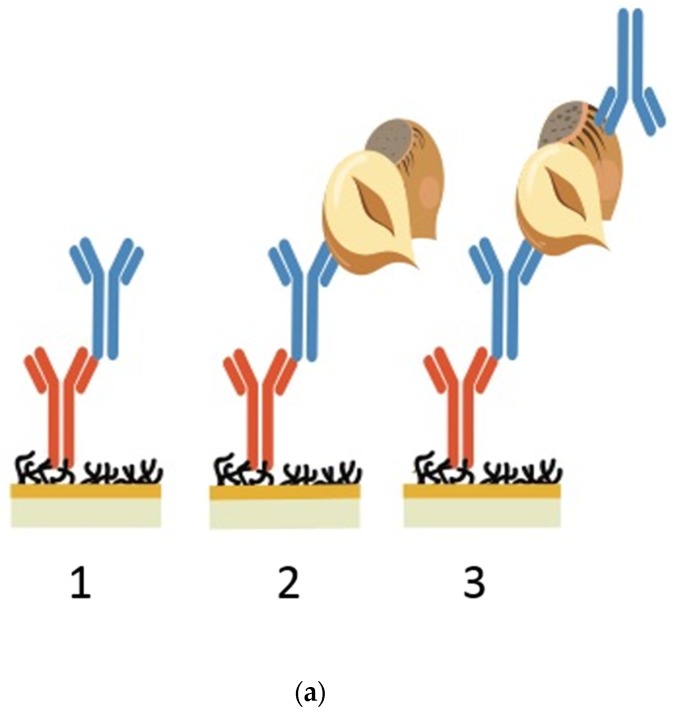

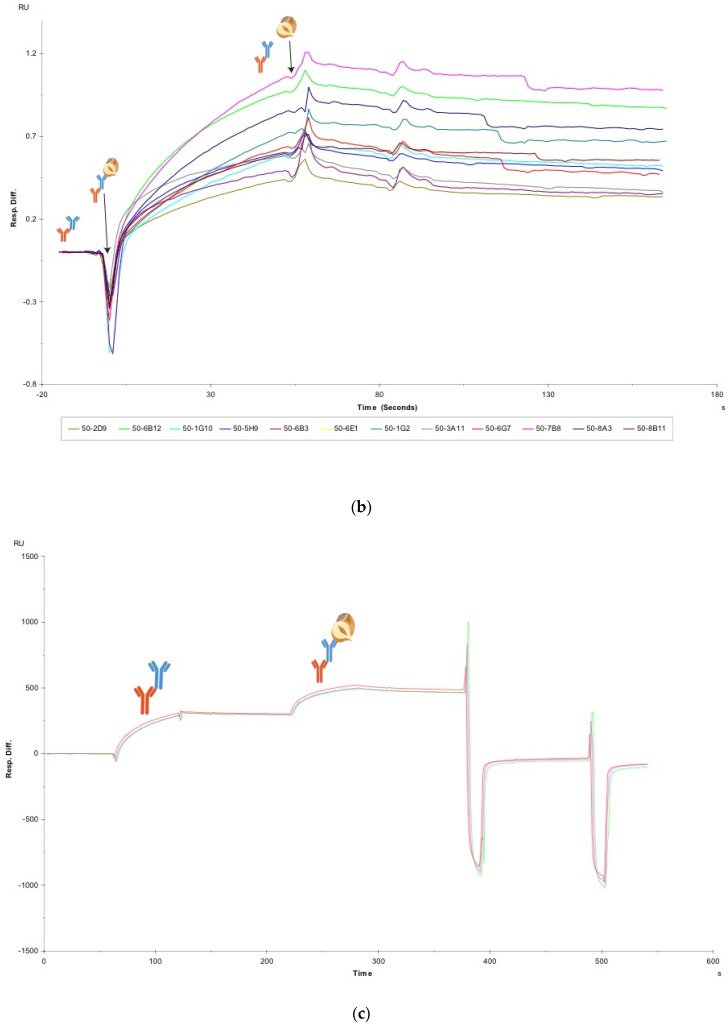
(**a**) SPR screening assay for crude antibodies. The first image shows the capture of a crude anti-hazelnut mAb (blue) via its FC region by the FC specific IgG (orange). The second image shows the binding of total hazelnut protein to the anti-hazelnut mAb. The third image displays the sandwich pairing of an anti-hazelnut mAb (blue) towards hazelnut and another anti-hazelnut mAb (blue). (**b**) Normalised SPR sensorgrams for 12 crude antibody preparations against 20 ppm hazelnut extract. The hazelnut injection is indicated by the first arrow, which is followed by the association of the crude antibodies towards hazelnut. The second arrow indicates the start of the hazelnut dissociation from the antibodies. (**c**) Full sensorgram of a crude antibody towards hazelnut in triplicate. The first curve shows the capture of the crude antibody via its FC region, the second curve the binding of hazelnut to that antibody and the following two spikes the regeneration.

As the antibodies being screened for this study were in an un-purified, crude form, a capture method was used to allow for on-chip purification and proper orientation of anti-hazelnut mAbs (see Figure 1a). Although the FC-IgG itself may have suffered with orientation issues because a sufficiently high density was immobilised, these concerns could be alleviated as there was still a significant proportion of correctly orientated FC-IgG. Furthermore, the FC-IgG surface allows for the anti-hazelnut mAbs to be captured predominantly in a ‘tail-on’ orientation, exposing their unoccupied antigen binding sites [30]. The FC-IgG was immobilized in flow cells 2–4 (flow cell 1 was left blank as a reference surface) to create a homogeneous surface. Then, the crude antibody sample was injected for capture by the immobilized FC-IgG. Following this, the hazelnut extract was injected and allowed to bind with the captured crude antibody sample. Each cycle was performed in duplicate. The duplicate results, across different flow cells, were used to determine the reproducibility of analyte binding levels. The captured antibody/hazelnut complex was completely removed from the FC-IgG specific surface before injecting the next crude antibody sample. 

A key benefit of SPR is the ability to re-use the sensor chips. Proper surface regeneration was achieved using standard conditions. These regeneration conditions removed the captured antibody/hazelnut leaving the FC-IgG surface intact. The signal after regeneration only resulted in a slight loss of baseline response, but subsequent antibody/analyte injections were able to reach response levels within ± 10% of the original response levels. Every few cycles, some antibodies were re-injected to ensure that the same levels and binding ratios could be reproduced; for example, S-50-5H9 was re-injected between other antibodies and was able to bind to hazelnut at 87.63, 92.22 and 94.93 RU. For the cross-reactivity study, sometimes only one regeneration pulse was required due to less antigen binding and therefore less protein to remove from the surface.

The overlay plot presented in Figure 1b displays sensorgrams with the association curves for 12 different crude antibody preparations against hazelnut (compare with Supplementary Material Figure S3 for duplicate curve reproducibility across two flow cells). As dissociation is not of primary concern in LFIA, this characteristic was not focused on here. Each sensorgram composed of the crude antibody capture step, followed by the injection of the hazelnut extract and then the subsequent surface regeneration. An example of the full sensorgram before data processing can be seen in Figure 1c (data for 1 antibody, overlaid in triplicate) where the first curve represents the capture of the hazelnut antibody, the following curve the binding of the antibody with hazelnut and the subsequent spikes, the standard regeneration conditions. The levels for crude antibody capture ranged from 40-160 RU and the antigen binding response ranged from 20-130 RU; these responses are in correspondence with the range of levels reached in [11]. The binding curves were normalized as described in the methods section. From Figure 1b, a visual interpretation of the association rates of the crude antibodies can be made. The start of the association phase is indicated by the first arrow (Figure 1b). Those antibodies with a steeper slope incline at the dip (e.g., 50-7B8) have a faster association towards hazelnut compared with the antibodies with a shallower curve (e.g., 50-2D9). The visual interpretation of the curves was confirmed by slope analysis in Microsoft Excel and was reproducible across two separate cycles in two different flow cells. The crude mAbs were ranked based primarily on association rates (visually and confirmed in Excel) and subsequently on hazelnut binding plateau values as can be seen in Table 1. As this study aimed for a quick and simple SPR screening method, no attempt was made to compare the absolute association, dissociation and equilibrium constants of the crude antibodies.

Although the main purpose of this screening method was to select ultra-fast antibodies for a high-speed LFIA, it is also necessary that these antibodies exhibit good sensitivity. Therefore, the antibodies were grouped first according to their association speeds towards hazelnut and then according to the amount of hazelnut that they were able to bind (Table 1). Regardless of the extent of hazelnut binding, the most desirable parameter in this study was the speed of mAb to hazelnut binding for final application in LFIA. The experiments were performed in duplicate with identical results. According to this ranking, the three best (fast and able to bind most hazelnut) antibodies selected were 50-7B8, 50-6B12 and 50-8A3. The antibody which was able to bind the least and had the slowest association toward hazelnut was 50-2D9 with the second and third slowest being 50-3A11 and 50-5H9, respectively. Even those crude antibody preparations at the bottom of the table were still capable of binding sufficient hazelnut, meaning that even the less optimal mAbs could be applied as capture ligands in a direct SPR assay. 

#### 3.1.1. Cross Reactivity 

The cross reactivity study was carried out with the top two fastest (50-7B8 & 50-6B12) and the two slowest (50-3A11 & 50-2D9) crude antibodies (listed in Table 1). The percentage of cross reactivity was determined by dividing the binding response (RU) of the tree nut/peanut extract by the corresponding binding response of hazelnut toward that particular crude antibody (see Supplementary Material 4, Table S1). The fastest antibody (50-7B8) cross reacted with walnut at 17%, making it unsuitable for application in a hazelnut LFIA. The second fastest (F) antibody (F-50-6B12) exhibited no significant cross-reactivity toward the tested tree nut/peanut extracts, so this antibody was selected for further testing for use as the ‘best’ antibody for the LFIA prototype. Both of the slowest antibodies (50-3A11 & 50-2D9) displayed significant cross-reactivity towards multiple other tree nut/peanut extracts and were capable of binding less hazelnut, making these antibodies unsuitable for LFIA. Therefore, the third slowest (S) antibody (S-50-5H9) was also tested for cross-reactivity and it was found that it did not exhibit significant cross-reactivity toward the tested tree nut/peanut extracts and so was carried forward as the less optimal antibody for LFIA prototyping. 

#### 3.1.2. Sandwich Pairing 

A different antibody was captured in each of 3 flow cells, leaving flow cell 1 blank as a reference. Hazelnut extract was injected simultaneously over all flow cells, meaning that three hazelnut binding curves were generated per cycle. Succeeding this, one crude antibody was injected simultaneously over all flow cells, attaining sandwich pair information against itself, and against two other crude antibodies. 

**Figure 2:**
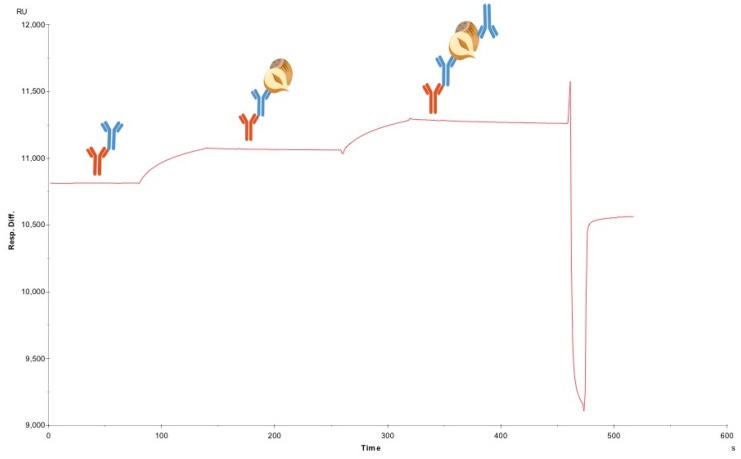
SPR based sandwich pairing. Sensorgram depicting crude “good” sandwich pair F-50-6B12 + F-50-6B12. The first curve in the sensorgram represents the hazelnut binding to F-50-6B12. The following curve shows the binding of a second F-50-6B12 to the hazelnut protein extract.

This method was repeated for all the antibodies to be tested for sandwich pairing. The lack of binding of secondary mAbs, when there was no hazelnut protein bound to the capture mAbs, demonstrated the absence of unwanted binding to unoccupied FC-IgG in the flow cell. Consequently, when binding did occur, following hazelnut injection, this confirmed the formation of a sandwich pair. Although F-50-6B12 and S-50-5H9 could form sandwich pairs both with one another and some of the other antibodies, the most successful pairs (able to bind the most hazelnut and subsequent antibody) were with themselves. In Figure 2. the sandwich pairing for F-50-6B12 and itself can be seen. In this sensorgram, the first curve represents hazelnut binding with F-50-6B12 and the subsequent curve shows the binding of F-50-6B12, indicating that F-50-6B12 is capable of binding to two distinct epitopes and can form a sufficient sandwich pair. Furthermore, it appears that F-50-6B12 has very little dissociation, although this is not necessarily an important characteristic within LFIA, it is indicative of the formation of a stable sandwich pair. The sandwich binding of S-50-5H9 and itself can be seen in the Supplementary Material Figure S5. As the optimal antibody (F-50-6B12) and the less optimal antibody (S-50-5H9) were capable of forming sandwich pairs with themselves, only these antibody preparations were finally purified for application in a LFIA prototype.

### 3.2. Lateral Flow Immunoassay Prototypes

First, the optimal mAb test line concentration was determined using the purified antibodies. In order to make a fair comparison between the two antibodies it was necessary to use the same dispensing conditions for each. It was found that the strips with a 0.2 mg/mL mAb at the test line gave a background response in a blank matrix for S-50-5H9, so this concentration was rejected. The 0.05 mg/mL test line strips suffered from a loss of sensitivity for both antibodies. The 0.1 mg/mL test line strips gave no response in the blank, but were not as sensitive. Therefore, the optimum test line condition for both mAbs was found to be 0.15 mg/mL. Different control line concentrations were also tested, with the optimal concentration being 0.1 mg/mL. This concentration was selected as it still gave a significant control line response without causing a background response in a blank. 

**Figure 3:**
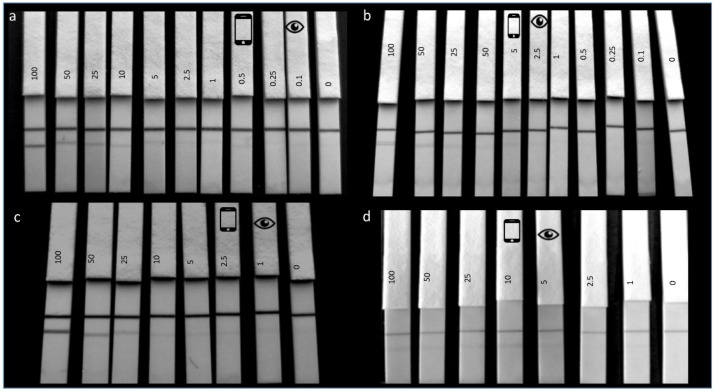
Lateral flow immunoassay limit of detection experiments. (**a**), F-50-6B12, (**b**), S-50-5H9: LFIAs showing the LOD determination of hazelnut protein extract spiked in PBS in the range of 100 ppm to 0.1 ppm with the last LFIA being a blank (0 ppm). In all LFIAs, the upper line is the control line and the lower line the test line. The visual LOD is indicated by the eye icon and the detection limit using a smartphone camera is indicated by the smartphone icon. (**c**), F-50-6B12, (**d**), S-50-5H9: LFIAs showing matrix LOD of hazelnut protein extract spiked in blank cookie extract (1:100 in running buffer) in the range of 100 ppm to 1 ppm (with the last strip representing a blank 0 ppm). The visual LOD is indicated by the eye icon and the detection limit using a smartphone camera is indicated by the smartphone icon.

For the optimal antibody (F-50-6B12), an LOD of 0.1 ppm for hazelnut protein extract in spiked buffer was achieved and for the less-optimal antibody (S-50-5H9), an LOD of 2.5 ppm was reached (Figure 3a,b). The results are consistent with the observations made in the SPR experiments, as F-50-6B12 was capable of binding more hazelnut compared with S-50-5H9. As can be seen in Figure 3a,b, the naked eye is able to read at a lower limit (visual LOD indicated by the eye icon) compared with the smartphone camera (smartphone LOD indicated by smartphone icon), this is likely owing to ambient light conditions which come into effect when recording the smartphone image. The spiked buffer experiments were reproducible across different days (*n* = 3) with identical visual LOD’s being reached for each repetition. As the smartphone images were recorded over different days and times, with no light control mechanism, differences are observed in the ambient lighting conditions in the images. 

**Figure 4:**
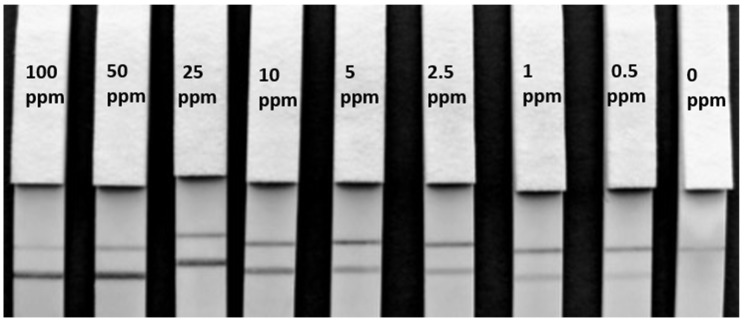
F- 50-6B12 Lateral flow immunoassay matrix limit of detection. Lateral flow strips for F-50-6B12 showing the matrix LOD of hazelnut protein extract spiked in blank cookie using 50 µL spiked sample and 50 µL RB. A clear LOD of below 0.5 ppm can be visualized both with the naked eye and with a smartphone camera.

To understand the LFIAs applicability to real life samples, the matrix LOD’s were subsequently determined by spiking hazelnut extract into a blank cookie extract. When using 1µL of spiked cookie extract in 100 µL of RB, a matrix LOD of 1 ppm could be achieved for F-50-6B12 (Figure 3c.) and of 5 ppm for S-50-5H9 (Figure 3d). As a much lower LOD was achieved for F-50-6B12 in the spiked buffer experiments, the matrix LOD experiments were repeated using 50 µL of spiked cookie extract (in RB) and 50 µL of RB in order to try and increase the sensitivity of the LFIA. For the less optimal mAb (S-50-5H9), these assay conditions resulted in a false positive, with even the blank producing a test line signal. However, under these conditions, F-50-6B12 was easily able to detect below 0.5 ppm (see Figure 4), making it the most sensitive hazelnut LFIA currently reported. The lowest LOD in spiked matrix for commercially available hazelnut LFIAs is currently 1 ppm [4]. This means that the LFIA prototype for the optimal mAb developed in this study is equally or even more sensitive than the currently reported LFIAs, even before any further optimization. 

To further exemplify future use in real life, the F-50-6B12 LFIA prototype was also tested in a decreasing amount of commercial hazelnut cookie extract, diluted in a blank cookie extract. In this way, F-50-6B12 was still able to detect the presence of the hazelnut cookie even when it was diluted by 10^6^ in a blank cookie. 

In order to determine the kinetics of the LFIAs, the strips were tested in a high concentration of hazelnut (50 ppm) and the timing of the appearance of the test line was recorded. Although the test line kinetics were the same when using lower/higher concentration of total hazelnut protein, the appearance of the test line was easier to distinguish when using a higher concentration, making it possible to more accurately record the timing of the line appearance. First, the kinetics were determined for each LFIA batch individually, across different days (*n* = 3), to establish an average visual response time and standard deviation (*n* = 8) for the test line appearance. Subsequently, the two LFIAs were one-to-one compared for the speed of the formation of the test lines which was recorded by video using a smartphone recording (Supplementary Material 6: Figure S6 smartphone video screenshots for video recording see Video S1). In Supplementary Material S6. a kinetic comparison between the two different LFIAs is demonstrated. By making time-resolved screenshots from a smartphone video recording (every 5 s) it is possible to distinguish the appearance of the test line of the F-50-6B12 LFIA at a much earlier (30 s) stage than the appearance of the test line for the S-50-5H9 LFIA (60 s). In reality, it is possible to distinguish the test line slightly earlier with the naked eye, compared with the smartphone recording. Therefore, visually the test line for F-50-6B12 first appeared, on average, at 30 s with a standard deviation of ± 1.2 s. The test line for S-50-5H9 appeared on average at 52 s with a standard deviation of ± 2.2 s. The LFIA kinetic results are in direct agreement with the results from the SPR experiments, where F-50-6B12 also exhibited nearly 2 × faster association with hazelnut compared with S-50-5H9 (see Table 1; slope analysis). The F-50-6B12 strips could easily be read visually or with a smartphone camera within 2 min and even the S-50-5H9 strips could be read within 5 min. 

### 3.3. Smartphone Detection

**Figure 5:**
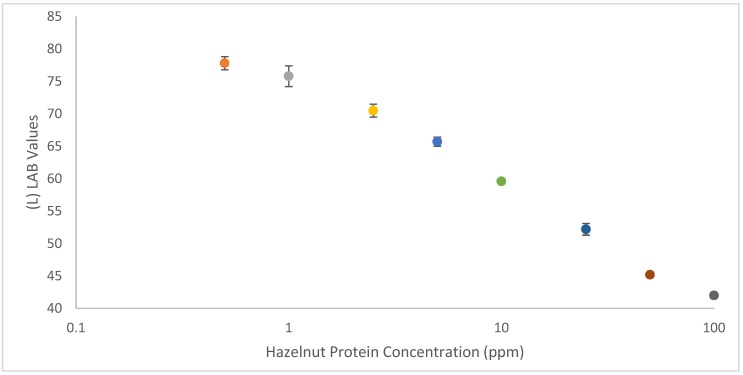
A calibration curve showing the relationship between (L)LAB values of test lines of hazelnut LFIA in a decreasing concentration of hazelnut protein (in blank cookie). Error bars have been included to show the standard deviation across multiple (*n* = 3) measurements. An (L)LAB value of 100, (0, 0) corresponds to a true white and of 0 (0,0) to a true black, in this study the lowest L value was 42 and so the L (LAB) axis begins at 40.

The majority of smartphone-based lateral flow readers rely on related assay-specific developed apps [31,32]. These apps can be used to semi-quantify LFIAs by establishing a calibration curve based on color values for test lines of LFIAs versus analyte concentrations. In the same way, color values can be determined using freely downloadable apps from Google Play Store. More researchers are switching to cieLAB/LAB color space analysis, as it has a more extensive color range (gamut), which more accurately represents how humans visually interpret colors and therefore, is device independent. Like RGB, LAB values are composed from three criteria, the L represents luminosity and A and B represent color space; unlike RGB only the L value provides information about the darkness/lightness of the selected region. Using the (L)LAB values obtained from the test lines, it was simple to establish a calibration curve to semi-quantify the strip tests by plotting total hazelnut protein concentration (in blank cookie) against (L)LAB values (see Figure 5 below). Background measurements were made from under the test line region on all of the strips, as at this stage a light box was not used to control the ambient lighting conditions of the photos. There is a clear relationship between the (L)LAB values and the concentration of hazelnut present in the sample, with lower hazelnut concentrations corresponding to higher (L)LAB values. The applied method did not utilize any light-box or dedicated algorithm to control ambient lighting conditions, indicating that it is possible to use a smartphone to semi-quantify carbon nanoparticle-based LFIAs without attachments. In this way, it is possible for anybody to perform their own smartphone analysis using only an LFIA calibration range and freely downloadable apps. 

## 4. Discussion

Surface plasmon resonance was used to screen antibodies in their un-purified state based on their fast association, specificity and sensitivity towards hazelnut, for use in LFIA. This method saves significant time and resources compared with selecting mAbs by ELISA. In ELISA, it is preferred to use purified mAbs and the antibody purification process takes approximately one day for each antibody. Considering that in this study, 12 mAbs were ranked by SPR as an analysis tool, if these would have first needed purification, it would have taken over a week longer to get to the antibody assessment stage. As the method only requires small volumes of un-purified mAbs, it is possible to start assessing the mAb characteristics as early as the fusion stage. Additionally, as SPR is a label-free technique, even more time is saved by not having to perform additional labelling experiments, and more unequivocal information is obtained from SPR compared with ELISA. 

The SPR results made it possible to select a very good and a less optimal antibody pair for application and comparison in a high-speed LFIA. The two prototype LFIAs displayed a significant difference in the timing of the appearance of the test line, with F-50-6B12’s test line appearing at least 20 s before the appearance of the test line on the S-50-5H9 strip. When considering moving towards consumer friendly food allergen detection, it is desirable to have LFIAs that give accurate, positive results, as quickly as possible, so that food can rapidly be assessed before its consumption. Quick allergen analysis can prevent unnecessary allergic reactions by allowing consumers to determine which portions of foods are safe to eat and which should be avoided. The proposed screening method could be extremely useful when trying to select antibodies of similar kinetics to use in a multiplex assay. In this way it would be possible to select capture/detector mAbs for a range of targets which have similar association rates towards their targets, so that when they are utilised in a multiplex assay, the T-lines appear within a similar temporal resolution. The optimal F-50-6B12 strips were able to detect the presence of hazelnut at trace levels in spiked buffer, spiked commodity and a real life hazelnut cookie, highlighting the LFIAs usefulness in real life. The F-50-6B12 LFIA is sensitive enough to protect even for the most sensitive hazelnut allergic individuals. Finally, a semi-quantitative smartphone readout was achieved by using simple and free color analysis apps to obtain device independent LAB values. This proves that even in the absence of additional light-control mechanisms, 3D-printed attachments and dedicated software apps, it is possible for anyone to obtain semi-quantitative LFIA results using their smartphones, provided that mAbs are labelled with carbon nanoparticles. Such apps could also be used to semi-quantify a multiplex assay. This study demonstrates a generically applicable proof-of-concept method for a novel association and sensitivity-based antibody selection procedure that can be applied to crude preparations for consequent application in LFIA with a visual or smartphone readout and an LOD in the low ppm range. 

## Figures and Tables

**Table 1:** Antibody ranking based on the visually observed association rates towards hazelnut, the confirmation by slope analysis in Excel and the amount of hazelnut bound (according to RU values observed in SPR).

Fastest Association (Visual)	Slope Analysis (Excel)	Maximum Hazelnut Plateau
50-7B8	0.0233	50-7B8
50-6B12	0.0215	50-6B12
50-8A3	0.0193	50-8A3
50-1G10	0.0174	50-1G2
50-1G2	0.0166	50-6E1
50-6G7	0.0155	50-6G7
50-6E1	0.0153	50-1G10
50-6B3	0.0145	50-5H9
50-8B11	0.0137	50-8B11
50-5H9	0.0114	50-6B3
50-3A11	0.0110	50-3A11
50-2D9	0.0109	50-2D9

## Supplementary Materials

The following are available online at http://www.mdpi.com/2079-6374/8/4/130/s1, Figure S1: Scanning electron microscope (SEM) image of the carbon nanoparticles conjugated to 50-6B12, Figure S2: SEM image of HF13502XSS nitrocellulose membrane, Figure S3: Overlay sensorgrams of 12 different antibodies towards hazelnut, Figure S4: Table S1, Displaying the percentage of cross-reactivity of different anti-hazelnut antibodies towards different tree-nut allergen extracts, Figure S5: Sensorgram depicting the sandwich pairing between 50-5H9 and itself, where the first curve represents the capture of 50-5H9, and the second curve the binding of hazelnut towards 50-5H9 and the third the subsequent binding of 50-5H9, Figure S6: Screenshots from smartphone video recording made at 5 second intervals, Video S1: Smartphone video recording of the 50-6B12 and 50-5H9 strips, where the development of the test line appears much faster for 50-6B12 compared with 50-5H9.
