# Supplementary material for: Rapid Antibody Selection Using Surface Plasmon Resonance for High-Speed and Sensitive Hazelnut Lateral Flow Prototypes
Source: Biosensors (Basel). 2018 Dec 14;8(4):130. doi: 10.3390/bios8040130 (PMC6316566; doi:10.3390/bios8040130)
Supplement: Supplementary file 1 [file biosensors-08-00130-s001.zip › FINAL REVISED BIOSENSORS SI.pdf]

# Supplementary Material:

# Rapid antibody selection using surface plasmon resonance for high-speed & sensitive hazelnut lateral flow prototypes

**Georgina M.S. Ross[1], Maria G.E.G. Bremer[1], Jan H. Wichers[2], Aart van Amerongen[2], Michel W.F. Nielen[1,3]**

*[1]RIKILT, Wageningen University & Research. P.O Box 230, 6700 AE Wageningen, The Netherlands.*

*[2] Wageningen Food & Biobased Research, BioSensing & Diagnostics, Wageningen University & Research, P.O Box 17, 6700 AA, Wageningen, The Netherlands*

*[3] Wageningen University, Laboratory of Organic Chemistry, Helix Building 124, Stippeneng 4. 6708 WE Wageningen, The Netherlands.*

*Email: georgina.ross@wur.nl Phone Number: +31 (3174) 84358

**Supplementary Material 1**. Scanning electron microscope (SEM) image of the carbon nanoparticles conjugated to 50-6B12. The images were made by drying a suspension of (1 in 5 dilution of conjugate in 100 mM borate buffer) carbon-50-6B12 onto a Millipore polycarbonate GTTP filter (nominal pore size 0.1 µm) and sputtering it with a fine coating of gold. The SEM conditions were a charge of 12 kV and a magnification of x 8,000. The conjugates are represented by the white grape like structures. A 0.1 µm pore had been circled in red to better indicate the scale.

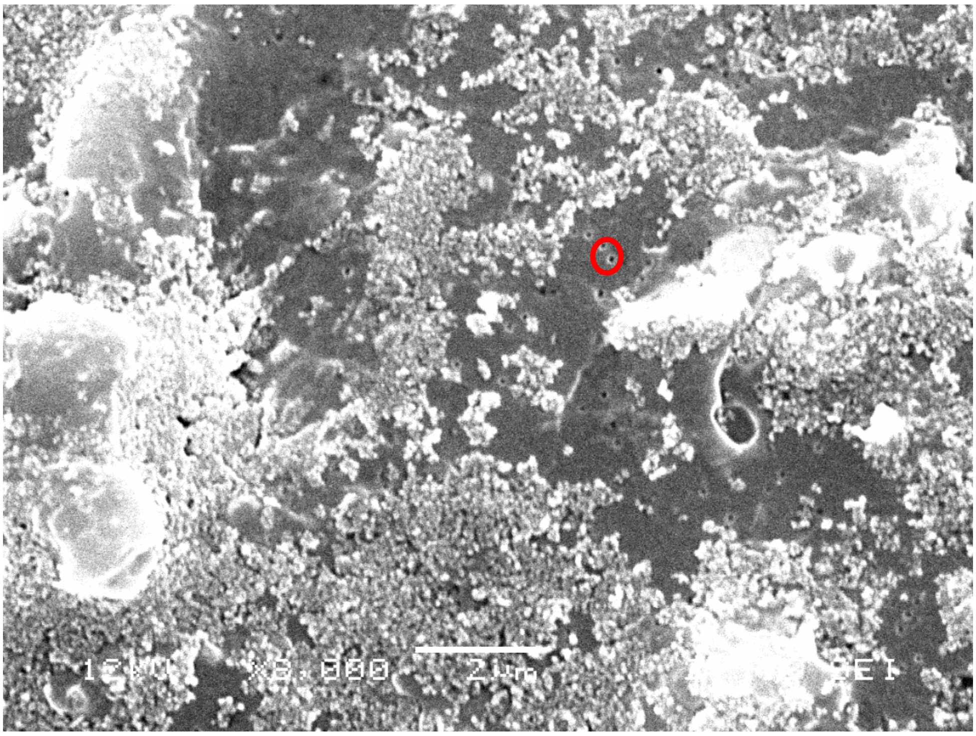


**Supplementary Material 2**: SEM image of HF13502XSS nitrocellulose membrane. The SEM conditions for this image were a charge of 6 kV and a magnification of x2,200.

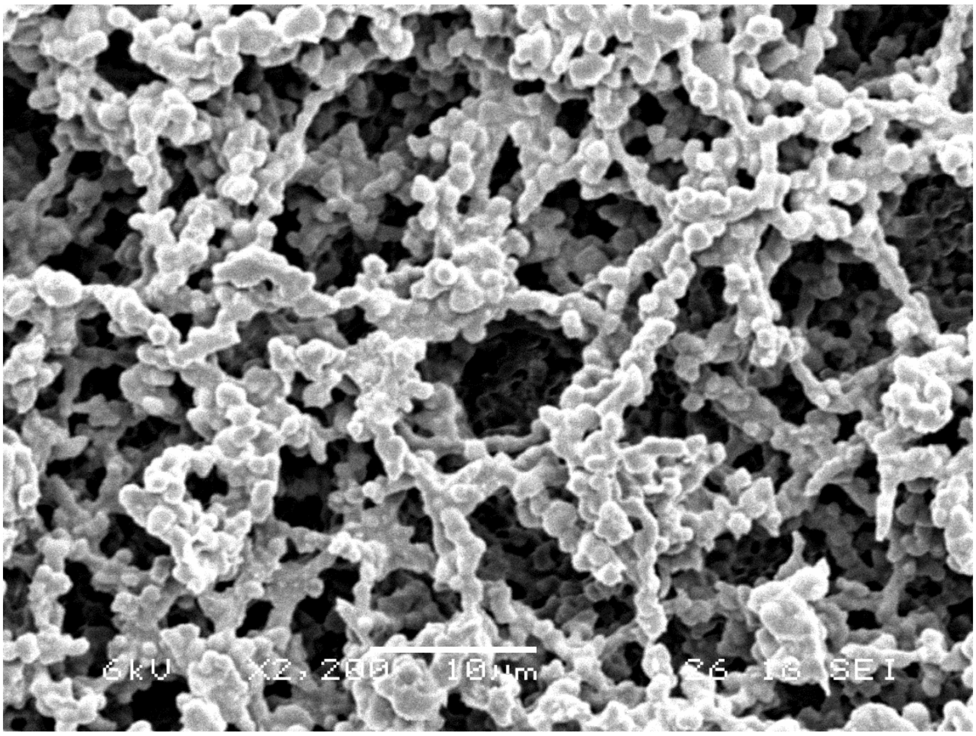


**Supplementary Material 3**. Overlay sensorgrams of 12 different hazelnut antibodies towards hazelnut.

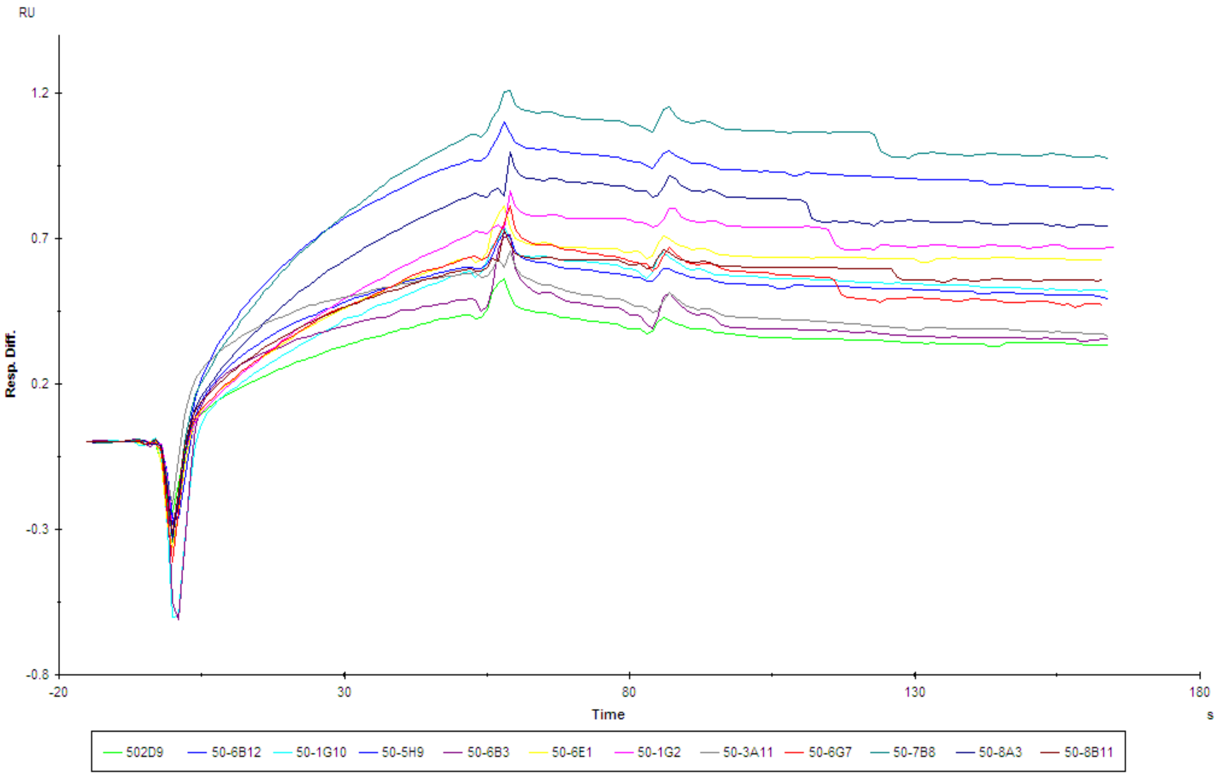


**Supplementary Material 4**. Table 1, Displaying the percentage of cross-reactivity of different anti-hazelnut antibodies towards different tree-nut allergen extracts. The percentage of cross reactivity was

determined by dividing the binding response (RU) of the tree nut/peanut extract by the corresponding binding response of hazelnut extract toward that particular crude antibody (%).

| mAb | Peanut | Pecan | Cashew | Almond | Walnut |
|---|---|---|---|---|---|
| 50-7B8 | N/A | 0 | N/A | 0 | 17 |
| 50-6B12 | N/A | 4.5 | N/A | 0 | 3 |
| 50-5H9 | N/A | 4.7 | N/A | 2 | 4.5 |
| 50-3A11 | N/A | 17 | N/A | 1.7 | 13 |
| 50-2D9 | N/A | 42 | N/A | 4 | 125 |

**Supplementary Material 5.** Sensorgram depicting the sandwich pairing between 50-5H9 and itself, where the first curve represents the capture of 50-5H9, and the second curve the binding of hazelnut towards 50-5H9 and the third the subsequent binding of 50-5H9.

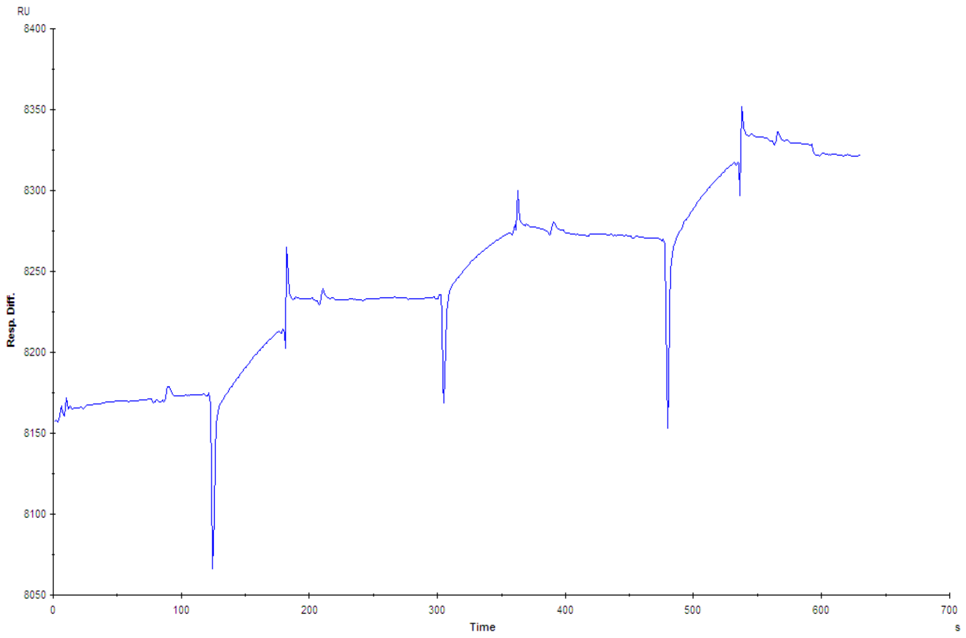


**Supplementary Material 6:** Screenshots from smartphone video recording made at 5 second intervals. Lateral flow immunoassay kinetic experiments. Time resolved photos of the appearance of the test and control lines on F-50-6B12 (red) and S-50-5H9 (yellow) LFIA strips. Screen shots taken from the smartphone video recording at 5 second intervals. A clear positive result can be seen for the F-50-6B12 strips within 30 seconds (indicated by red arrow) whilst a positive result for S-50-5H9 can only be seen after 60 seconds (indicated by yellow arrow).

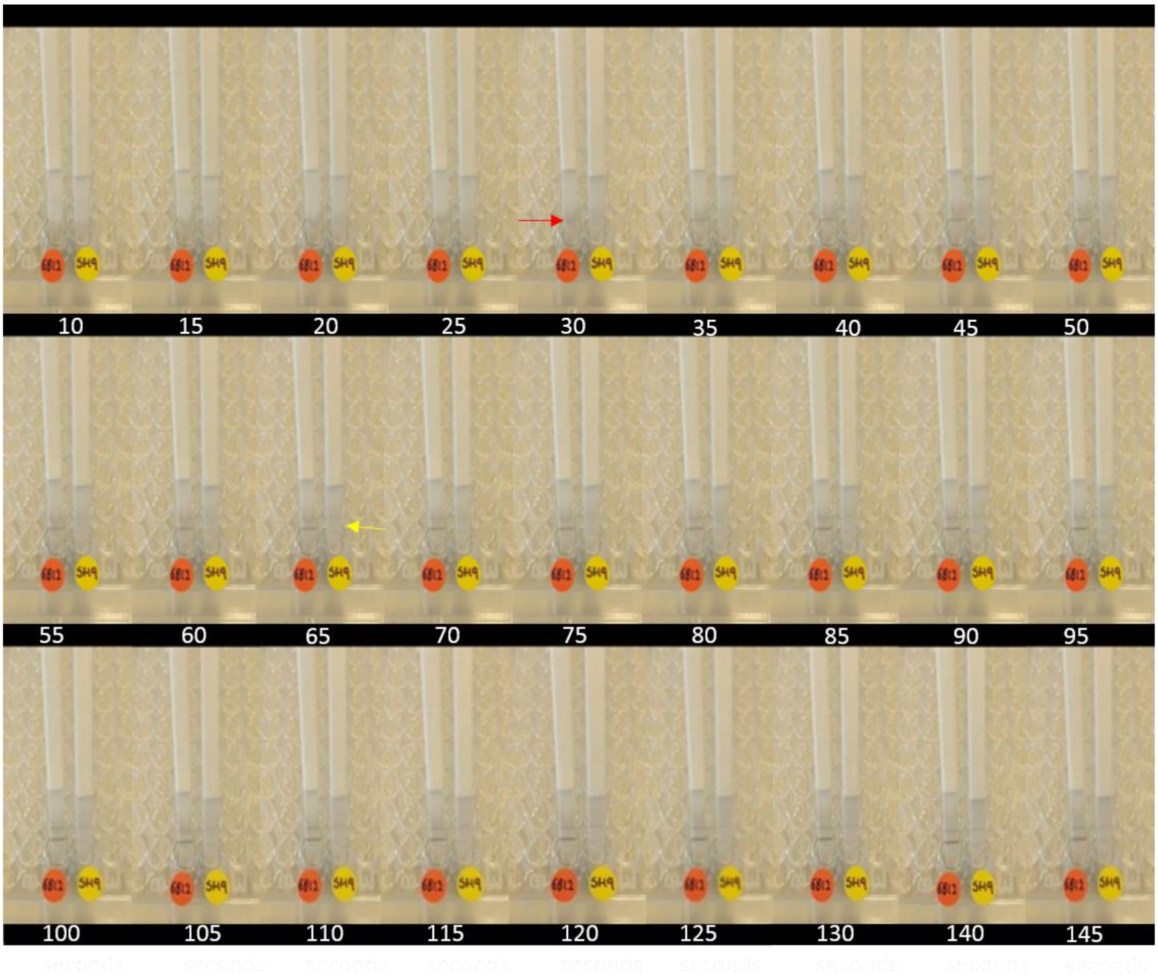


**Video S1.** Smartphone video recording of the 50-6B12 and 50-5H9 strips, where the development of the test line appears much faster for 50-6B12 compared with 50-5H9.

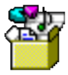

50-6B12 v 50-5H9
LFIA.mp4

https://drive.google.com/file/d/1IR3zFS5k6Eo1SUbqgzuU5l3i6S2_x_8r/view

Still for video:

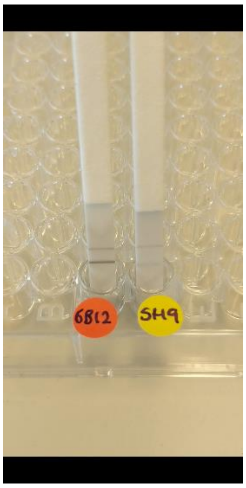


**Funding**

This project has received funding from the European Union’s Horizon 2020 research and innovation program under the Marie-Sklodowska-Curie grant agreement No 720325, FoodSmartphone.
