# Supplementary figures and images for: Rapid Antibody Selection Using Surface Plasmon Resonance for High-Speed and Sensitive Hazelnut Lateral Flow Prototypes
Source: Biosensors (Basel). 2018 Dec 14;8(4):130. doi: 10.3390/bios8040130 (PMC6316566; doi:10.3390/bios8040130)

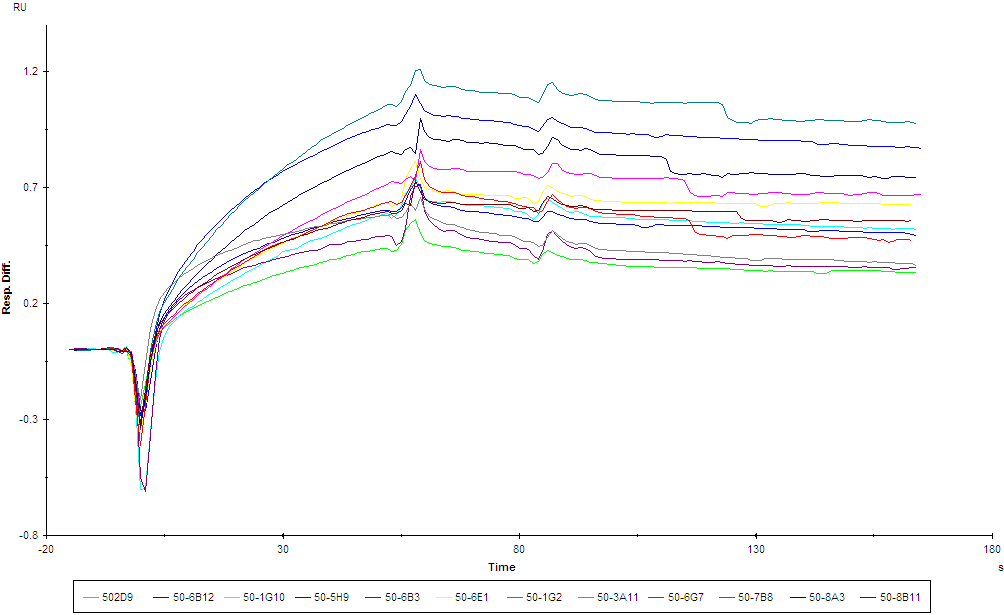

Supplement: Supplementary file 1 [file biosensors-08-00130-s001.zip › S3.tif]

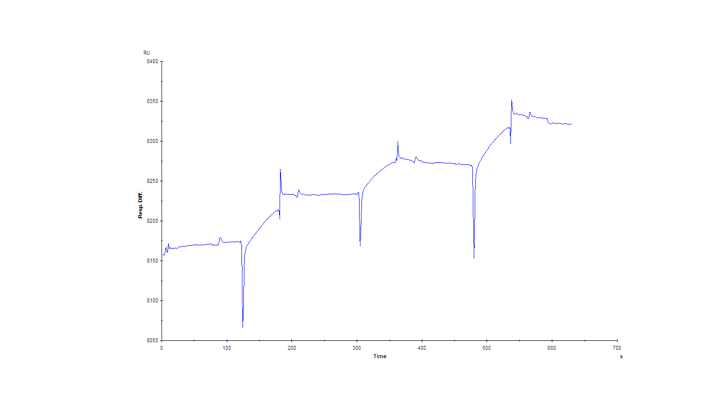

Supplement: Supplementary file 1 [file biosensors-08-00130-s001.zip › S5.tiff]

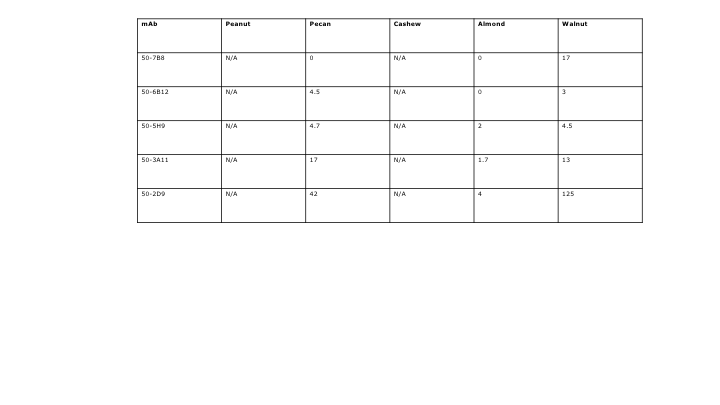

Supplement: Supplementary file 1 [file biosensors-08-00130-s001.zip › Supplementary material 4 Table S1.tiff]

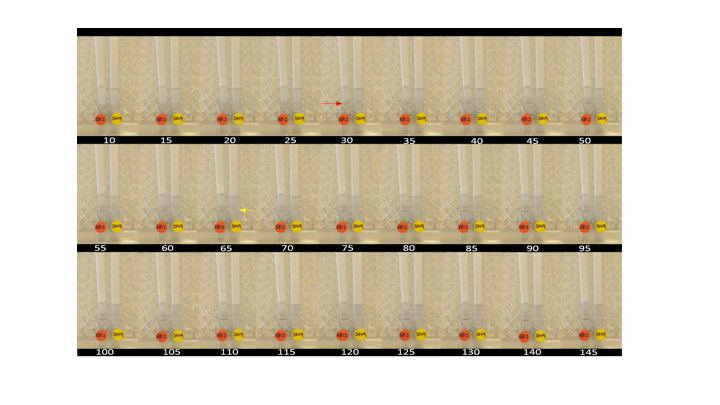

Supplement: Supplementary file 1 [file biosensors-08-00130-s001.zip › S6A.tiff]
